# Reliability of the Korean Version of the Eating and Drinking Ability Classification System in Children with Cerebral Palsy

**DOI:** 10.3390/children12060673

**Published:** 2025-05-23

**Authors:** Sangwon Hwang, Kang-Jae Jung, Yeonhee Choi, Dong-Wook Rha

**Affiliations:** 1 Department of Physical Medicine and Rehabilitation, Eulji University Hospital, Daejeon, 95 Dunsanseo-ro, Seo-gu, Daejeon 35233, Republic of Korea; sangwon_hwang@eulji.ac.kr (S.H.); jkj0925@eulji.ac.kr (K.-J.J.); 2 Department of Speech and Language Therapy, Eulji University Hospital, Daejeon, 95 Dunsanseo-ro, Seo-gu, Daejeon 35233, Republic of Korea; yeonheechoi@eulji.ac.kr; 3 Department of Rehabilitation Medicine, Severance Hospital, Research Institute of Rehabilitation Medicine, Yonsei University College of Medicine, 50 Yonsei-ro, Seodaemun-gu, Seoul 03722, Republic of Korea

**Keywords:** cerebral palsy, pediatrics, eating behavior, drinking behavior, efficiency, reproducibility of results

## Abstract

Background/Objectives: This study aimed to assess the reliability and validity of the Korean version of the Eating and Drinking Ability Classification System (EDACS) for children with cerebral palsy (CP). Methods: This was a prospective cross-sectional study conducted to psychometrically assess 40 children with CP. Reliability was evaluated by a physician in pediatric rehabilitation, a speech–language therapist (SaLT), and caregivers. Results: In the evaluation of each level and level of assistance in the EDACS, the agreement observed between the physician and speech therapist was almost perfect (κW = 0.940, 0.919). The agreement between the physician and the caregiver was substantial (κW = 0.618, 0.592), whereas it was moderate between the therapist and caregiver (κW = 0.557, 0.556). Intra-rater reliability remained almost perfect, with the physician (κW = 0.979), the SaLT (κW = 0.980), and caregivers (κW = 0.980). The EDACS showed moderate to high correlation with the Functional Oral Intake Scale, Gross Motor Function Classification System, Manual Ability Classification System, and Communication Function Classification System (Kτ = −0.863, 0.656, 0.720, 0.616). Conclusions: These findings support the Korean EDACS as a reliable tool for classifying eating and drinking abilities in children with CP, thereby enhancing its clinical utility and communication among non-professionals and professionals.

## 1. Introduction

Cerebral palsy (CP) is the most common developmental disorder in childhood, defined as a group of disorders characterized by motor dysfunction due to non-progressive lesions in the brain, which occur before birth or during infancy [1]. In addition to motor impairments resulting from brain injury, eating-related difficulties, including oromotor dysfunction and suspected swallowing problems, are among the more readily noticeable symptoms in clinical settings, compared with other abnormalities such as sensory deficits, cognitive impairments, and speech and language disorders. Swallowing difficulties, defined as problems with transporting food, liquid, or saliva from the oral cavity to the stomach, have been reported in approximately 50% of children with CP, whereas feeding difficulties—which encompass caregiver–child interactions beyond the act of swallowing itself—have a reported prevalence of approximately 54% [2]. If left untreated, eating and swallowing problems in children with CP may lead to nutritional deficiencies, failure to thrive, and respiratory problems such as recurrent aspiration pneumonia, which are closely associated with mortality [3,4].

The Eating and Drinking Ability Classification System (EDACS) was developed to assess and classify eating and drinking abilities in children with CP, aged ≥3 years, into five levels based on the key features of safety and efficiency [5,6]. Higher levels indicate more severe impairments in the ability to eat and drink. Additionally, based on the level of assistance required, children are further classified into three levels: independent, assistance required, and totally dependent. Better understanding of limitations in eating and drinking abilities can inform appropriate treatment strategies such as food and fluid texture modification, close monitoring of aspiration risk using videofluoroscopic studies, and interventions aimed at improving the overall safety and efficiency of oral intake. Recent studies have highlighted the role of EDACS-based assessment in guiding clinical decisions to reduce risks of respiratory problems, poor nutrition and hydration, and complications related to poor respiratory health and dental hygiene [7,8,9]. Along with EDACS, the Functional Oral Intake Scale (FOIS) was developed to measure oral intake in patients with stroke. The FOIS is a seven-level scale used to classify the extent to which individuals consume food and liquid orally. Levels range from tube-dependent feeding (level I) to full oral intake without restrictions (level VII), with intermediate levels representing various degrees of oral intake with modifications or supplements [10]. The FOIS provides a structured assessment of oral intake status, which complements the EDACS in evaluating both the functional and safety aspects of eating and drinking. It shows high reliability for pediatric swallowing disorders and has been used to assess the effectiveness of therapeutic interventions aimed at improving pediatric swallowing function [10,11,12].

The high inter-rater and intra-rater reliability of the original EDACS, as well as its translations into German, Dutch, and Chinese, has been demonstrated in previous studies [5,6,13,14]. The Korean version of the EDACS showed high reliability in comparisons between speech and language therapists (SaLTs) and caregivers of adult patients with CP [15]. However, research on pediatric CP has not yet been conducted. Moreover, there are concerns regarding agreement between non-professionals (i.e., caregivers) and professionals in determining classification levels.

Therefore, this study aimed to assess the inter-rater and intra-rater reliability of the Korean version of the EDACS among a physician, a SaLT, and caregivers. In addition, we examined its correlations with other functional classification systems—such as the FOIS, Gross Motor Function Classification System (GMFCS), Manual Ability Classification System (MACS), and Communication Function Classification System (CFCS)—to explore the relationship between eating and drinking abilities and overall functional status, and to assess the clinical utility of the EDACS in multidisciplinary care planning.

## 2. Materials and Methods

### 2.1. Participants

In this prospective cross-sectional study, we used a range of functional classification systems and measures to assess children with CP aged between 3 years and 18 years. Eligible participants were children who visited the Department of Rehabilitation Medicine at Eulji University Hospital in Daejeon between October 2022 and August 2023. Children whose eating habits and postures had changed owing to surgery within the past 3 months were excluded. This study proceeded only with cases where both the patient and caregiver held Korean nationality and were capable of communicating in Korean and provided informed consent. In this study, the required sample size was estimated using the PASS software (version 12, NCSS, LLC, Kaysville, UT, USA) based on the method described by Flack et al. (1988) for the two-rater kappa statistic [16]. The goal was to detect a substantial to almost perfect agreement (κ = 0.80) compared to a moderate level of agreement under the null hypothesis (κ = 0.50), with a significance level of 0.05 and a statistical power of 90% (β = 0.10). The interpretation of kappa values in our analysis follows the widely used guideline proposed by Landis and Koch (1977) [17]. Based on this calculation, and allowing for a 15% dropout rate, a total of 42 children with cerebral palsy were recruited. Ultimately, 36 participants were included in the final analysis, having been evaluated by three independent raters. Consent was obtained following face-to-face explanations by the research personnel. The same consent form was provided to both participants and their caregivers. For each participant, informed consent was obtained from both the child and their legal guardian. In addition, caregivers who participated as raters also provided informed consent. This study was approved by the Institutional Review Board of the Eulji University Hospital. It was supported by Eulji University in 2022.

### 2.2. Procedures

Caregivers were defined as adults aged 18 years or older who were either the child’s parent or a caregiver who had observed the child’s mealtime behavior for at least one week. Of the 40 caregivers, 38 were parents of the children, and 2 were non-parent caregivers (a private caregiver and a teacher). Explanatory materials describing the evaluation methods and assessment tools were provided to all caregivers. Basic information—including diagnosis, CP subtype, comorbidities, and seizure history—were collected. The physician was an experienced professional with over 5 years of experience in pediatric rehabilitation and regularly used these assessments in clinical practice. The SaLT also had over 5 years of experience treating children with CP and was familiar with EDACS. Prior to participant recruitment, the physician and the SaLT held a 20 min discussion to review the study protocol and confirm a shared understanding of the EDACS assessment procedure. Following consent acquisition, caregivers were provided with the Korean version of the EDACS and given more than one week to read and understand the materials. During this period, they were encouraged to ask questions, and clarification was offered as needed by the first author, a physician specializing in pediatric rehabilitation medicine. The physician evaluated the GMFCS and MACS through direct assessments, while the SaLT evaluated the CFCS through brief interviews with the participants for 5 min. Evaluation was conducted through observational assessment using video recordings and contextual information provided by caregivers. Two home-based video recordings, spaced 1–2 weeks apart, were conducted by caregivers. Each video included the child’s meal preparation setting, utensils used, and a 10 min recording of the eating process. In addition, caregivers submitted written annotations regarding the child’s post-meal oral status, including total mealtime duration, signs suggestive of aspiration (e.g., coughing), and the presence of excessive drooling. Food and fluid textures were chosen freely by the caregiver or parent, without specific instruction, in order to reflect the child’s typical eating and drinking environment. This video-based observational approach was developed with reference to previous studies using caregiver-recorded mealtime videos in children with CP [18,19]. Given their daily involvement, caregivers naturally possessed greater familiarity with the child’s typical mealtime behaviors than the other raters did. At the time of the video recording, caregivers were instructed to conduct the EDACS evaluation independently, without being supervised by a physician or a SaLT. Evaluations by the physician and the SaLT for the EDACS were conducted separately based on the submitted videos, with the physician additionally evaluating the FOIS. The same procedure was repeated using videos that were re-taken at intervals. The physician and the SaLT each evaluated all 40 patients, and each patient’s caregiver evaluated only their own child. All raters were blinded to the other’s assessment to control for bias. The Korean version of the EDACS was used as the official Korean version, translated by Kim et al., and downloaded from www.edacs.org (accessed on 19 April 2025) [20].

### 2.3. Statistics

Statistical methods were used to analyze basic information. Inter-rater reliability among examiners was assessed by comparing results between caregivers and SaLT, caregivers and a physician, and SaLT and a physician. Intra-rater reliability was assessed by comparing the results from the same examiner on two separate occasions. Weighted kappa (κ) was used to evaluate the reliability of EDACS values among the three examiners and intra-rater reliability. Kendall’s tau-b (Kτ) was used to assess the relationship between the EDACS with the FOIS, GMFCS, MACS, and CFCS.

According to Landis and Koch (1977), weighted kappa values between 0.401 and 0.600 indicate moderate agreement, values between 0.601 and 0.800 denote substantial agreement, and values between 0.801 and 1.000 represent almost perfect agreement [17]. Munro’s classification was used to interpret Kendall’s tau-b (Kτ) values, where correlation coefficients below 0.25 indicate little correlation, values between 0.26 and 0.49 denote low correlation, values between 0.50 and 0.69 represent moderate correlation, values between 0.70 and 0.89 indicate high correlation, and values between 0.89 and 1.00 represent very high correlation [6].

All *p*-values below 0.05 were considered statistically significant, and data analysis was performed using the statistical software SPSS version 27.0 (IBM, Armonk, NY, USA).

## 3. Results

### 3.1. Participant Characteristics

A total of 42 children were included in this study. Of these, two withdrew their consent. Therefore, 40 children were included in the final analysis. Table 1 summarizes the demographic and clinical characteristics of the participants. The cohort included children with a broad range of motor, manual, and communication abilities, with the GMFCS, MACS, and CFCS levels ranging from I to V. All participants were capable of oral feeding (FOIS ≥ IV), and spastic CP was the most common subtype. No participant was tube-dependent or had undergone tracheostomy.

### 3.2. Assessment of EDACS—Level

**Figure 1:**
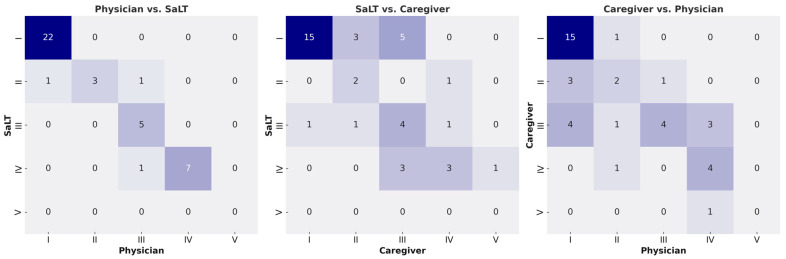
Heatmap of inter-rater agreement on EDACS levels between raters.

The reliability between the physician and the SaLT was almost perfect (κ = 0.940, 95% confidence interval (CI) 0.874–1.006 for the first, κ = 0.919, 95%CI 0.844–0.993 for the second assessment), with an absolute agreement in 37 cases (92.5%) and only 3 cases of discrepancies. The reliability between a SaLT and caregivers was moderate (κ = 0.557, 95%CI 0.374–0.740 for the first, κ = 0.556, 95%CI 0.379–0.733 for the second assessment), with absolute agreement in 24 cases (60.0%). Nevertheless, differences were observed in 16 and 17 cases in the first and second assessments, respectively. Similarly, the reliability between the physician and caregivers was moderate to substantial (κ = 0.618, 95%CI 0.450–0.785; *p* < 0.001 for the first assessment, κ = 0.592, 95%CI 0.431–0.753; *p* < 0.001 for the second assessments), with absolute agreement in 26 cases (65.0%). Among the disagreements observed, 62.5% of caregiver evaluations were lower than those of professionals. Nonetheless, complete agreement among all three evaluators was observed in 24 participants (60%) (Table 2 and Figure 1). EDACS level V was not assigned by either the physician or the SaLT in any of the cases, although one caregiver rated a child at level V. All children were capable of oral feeding (FOIS ≥ IV), and none were fully tube-dependent.

### 3.3. Assessment of EDACS—Level of Assistance

**Figure 2:**
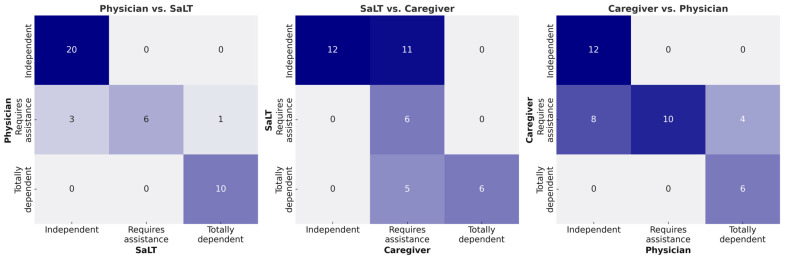
Heatmap of inter-rater agreement on EDACS level of assistance.

The reliability between the physician and the SaLT regarding the level of assistance needed was almost perfect for the first (κ = 0.887, 95%CI 0.780–0.994; *p* < 0.001) and second (κ = 0.859, 95%CI 0.739–0.979; *p* < 0.001) assessments. However, there were four participants whose evaluations did not match, accounting for 10% of the total. Of the 10 participants whom the physician evaluated as needing assistance, the SaLT made similar evaluations in six cases, differing by three evaluated as independent and one as completely dependent. The reliability between the SaLT and caregivers was moderate for the first (κ = 0.542, 95%CI 0.365–0.719; *p* < 0.001) and second (κ = 0.507, 95%CI 0.320–0.694; *p* < 0.001) assessments. Of the 22 participants that the caregivers evaluated as needing assistance, there was a complete agreement with those of the SaLT in 6 participants; the SaLT evaluated 11 participants as independent and 5 as needing complete assistance. The reliability between the physician and caregivers was moderate (κ = 0.636, 95%CI 0.461–0.812; *p* < 0.001) for both assessments. Similarly, of the 22 participants that the caregivers evaluated as needing assistance, complete agreement with the physician was found in 10 cases; the physician evaluated 8 participants as independent and 4 as needing complete assistance. Complete agreement among a physician, a SaLT, and caregivers was observed in 24 participants, accounting for 60% of all participants. In most disagreements, caregivers tended to evaluate the participants as needing less assistance. The maximum difference between assessments was one level (Table 3 and Figure 2).

### 3.4. Intra-Rater Reliability

The reliability was very high for each of the two assessments for the physician (κ = 0.979), the SaLT (κ = 0.980), and caregivers (κ = 0.980). The physician had discrepancies with two participants, while the SaLT and caregivers each had discrepancies with one participant. Moreover, the reliability for the level of assistance was perfect between the physician and caregivers with a SaLT, with a value of 1.0 across all participants (κ = 0.972). Almost perfect agreement was observed among all evaluators for both the levels and the level of assistance.

### 3.5. Assessments of EDACS Versus Other Classification Tools

Table 4 represents the distribution of the EDACS and other functional classifications of the participants. The validity between the FOIS assessed by physicians during the first EDACS assessment and the EDACS itself showed a significant negative correlation (Kτ = −0.863). Kendall’s tau values were 0.656 for GMFCS, 0.720 for MACS, and 0.616 for CFCS, indicating a high correlation with the MACS and a moderate correlation with the GMFCS and CFCS. The relationship between the EDACS levels of assistance and other CP functional assessments, such as the FOIS (Kτ = −0.595), GMFCS (Kτ = 0.629), MACS (Kτ = 0.741), and CFCS, was confirmed (Kτ = 0.353, Table 5).

## 4. Discussion

Our study confirmed a high level of agreement in the EDACS evaluations between professionals from different fields and also between professionals and non-professionals, responsible for attending to children with CP.

### 4.1. Inter and Intra-Rater Reliability

In this study, the inter-rater reliability of the EDACS level showed perfect agreement between professionals, including the physician and the SaLT. An absolute agreement was observed among 37 of the 40 participants. In contrast, moderate agreement was observed between professionals and caregivers. Notably, in cases of disagreement, caregivers—most of whom were parents—tended to rate participants’ EDACS levels lower than that by professionals: in 10 out of 15 cases compared to the physician, and in 11 out of 15 cases compared to the SaLT. This pattern is consistent with the findings from previous studies that suggested that evaluator familiarity and expertise influence EDACS scoring. For example, one study reported that parents rated their child’s eating and drinking function lower than professionals in only 4 of 52 cases (7.7%), with discrepancies of two or more levels occurring in just 1 case (2%) [6]. However, in this study, caregivers rated the function of participants lower than that of the physician and the SaLT in 10 out of 40 (25%) and 11 out of 40 (27.5%) cases, respectively, with discrepancies of two or more levels observed in 5 cases (12.5%) and 6 cases (15%). Notably, these discrepancies were concentrated at the lower end of the EDACS level: among the 10 physician–caregiver disagreements, the physician rated 7 children as level I, while caregivers rated them at level II or III; similarly, in 8 of the 11 SaLT–caregiver discrepancies, the SaLT assigned level I while caregivers rated the same child at level II or III. This pattern suggests that differences in developmental expectations, particularly regarding early independence in eating and drinking, may cause these disagreements. In East Asian cultures, where caregivers tend to provide prolonged support and often expect later attainment of independent self-feeding, parents may be more likely to perceive their child’s functional ability as limited, even when the child performs adequately according to the EDACS criteria [21,22]. Notably, children who had a two-level discrepancy in the EDACS were those with GMFCS and MACS levels of II or III, indicating relatively preserved function. However, they were in an ambiguous age range of 5–6 years, where independence in daily living had not yet been established. Notably, when the physician and SaLT assessed participants as EDACS level I, the caregivers evaluated the same participants as EDACS level III in four and five cases, respectively. Four of these cases were the same patients between the caregiver–physician and caregiver–SaLT comparisons. Considering cultural background, caregivers may assess these children with such ambiguous functional abilities at a lower functional level. Additionally, two of these participants were twins, and for the remaining two, while the physician and the SaLT assessed them as EDACS level I, with an assistance level of “independent”, the caregivers evaluated them as EDACS level III, with an assistance level of “requires assistance”. It is also possible that caregivers did not fully understand the EDACS or were overly cautious in their evaluations due to personal experiences. However, another explanation may be that caregivers had greater longitudinal exposure to their child’s mealtime difficulties—such as choking, fatigue, or food refusal—which may not have been visible in the limited video recordings.

Meanwhile, regarding the level of assistance, evaluations of professionals were more pronounced than were those of caregivers. Among 22 participants whom caregivers evaluated to require assistance, SaLTs evaluated 11 as independent, 5 as totally dependent, and only 6 as requiring assistance. Similarly, physicians evaluated eight participants as independent, four as totally dependent, and ten as requiring assistance. In some research, caregivers’ information may be more reliable than that of professionals who conduct one-time assessments [5,6,15]. In this study, the level of assistance can also be interpreted in the same context, as it is ultimately provided by the caregiver. Further research is needed to explore the trends in medical assessments conducted by both caregivers and professionals in the future.

The intra-rater reliability for EDACS levels and independence observed in this study was higher than that found in previous studies. In a study on the intra-rater reliability of eating assessments for pediatric CP, an interval ranging from 48 h to 2 months was adopted [14,19,23,24,25,26,27]. The interval set in this study was more than 1 week but less than 2 weeks for children with CP who regularly visited the hospital, trying to minimize memory bias and changes in patient health status. However, considering the storage period of food and ingredients during this interval, it is possible that the same or similar texture of foods was provided in both evaluations.

A meaningful aspect of this study is the participation of a physician as an expert evaluator. While the EDACS user instructions recommend involving someone who knows the child well and a professional with expertise in eating and drinking, the inclusion of a physician in this study provided added value given the clinical context of rehabilitation medicine, particularly in evaluating aspiration risks, guiding medical interventions, and coordinating team-based decision-making. In complex cases of CP, physicians are often responsible for initiating diagnostic procedures or medical interventions based on interdisciplinary input. Therefore, examining the level of agreement between the physician, therapists, and caregivers provides valuable information about the consistency of clinical judgments within a multidisciplinary rehabilitation team. However, previous studies have primarily involved expert evaluators such as occupational therapists and SaLTs, with limited participation by physicians as evaluators [6,13,14,15,27].

The strong reliability demonstrated in this study between the evaluations conducted by the physician, SaLT, and caregivers emphasizes the potential for effective coordination in the rehabilitation approach for children with CP. Such coordination facilitates appropriate and smooth communication across homes, rehabilitation facilities, and hospitals, ultimately maximizing the therapeutic outcomes related to the child’s feeding and drinking abilities.

### 4.2. Relationships Between EDACS and Other Functional Classification Tools

The correlation between various eating and swallowing assessment tools and the EDACS was reported to be considerable: the Dysphagia Severity Scale (Kτ = 0.74), Bogenhausener Dysphagie score 2 (Kτ = 0.79), and FOIS (Kτ = −0.346) [6,15,19]. Furthermore, the correlation between the MACS and EDACS is stronger than with the GMFCS, and this tendency is more pronounced in spastic CP [6,14,15,25].

The FOIS was originally designed to document the functional level of an individual’s daily oral intake, considering the need for swallowing compensation and dietary modifications in adults with acute stroke [10]. In the FOIS, higher scale numbers are closely related to normal eating. It has been used to assess the correlation with the EDACS for pediatric CP, demonstrating high inter-rater reliability [12].

In this study, a moderate or high correlation was observed for the EDACS compared with other CP functional classification systems. In particular, there was a strong correlation with FOIS, which is used to directly assess eating and drinking functions. Previous studies involving adults have shown that even with similar levels of swallowing function, the level of food intake may vary owing to social and personal factors, resulting in a low kappa value with the FOIS [6,15]. In this study, 21 patients were assessed as FOIS level VII and EDACS level I. Of the 22 patients assessed as EDACS level I, only 1 was FOIS level VI, and among the patients assessed as FOIS level VII, only 2 were EDACS level II. This contrasts with the findings in adult CP, where it was reported that individuals with similar swallowing functions may consume different types of food depending on their medical condition and personal environment [15]. However, pediatric patients with CP who have similar levels of eating function tend to consume comparable types of food.

Additionally, the relationship between EDACS level of assistance and the CFCS in this study was relatively low (Kτ = 0.353). The tendency to be relatively lower compared with other functional classification systems is consistent with previous findings [5,6,14]. In this study, there were eighteen patients classified as CFCS level V, but their EDACS levels varied: three were EDACS level I, six were EDACS level II, and nine were EDACS level III. These findings may be due to the CFCS’s focus on evaluating effective communication between a speaker and a listener, which does not directly assess oral function [28]. However, in this study, all participants who communicated verbally did so exclusively through speech rather than through augmentative or alternative communication methods. Therefore, within the context of this sample, CFCS levels may indirectly reflect aspects of oral function. Moreover, while oral–motor development primarily occurs between 10 and 30 months of age, the maturation of communication skills, including social interaction, occurs relatively later [6,29]. Therefore, their communication skills may be rated as low, while their eating functions may be relatively preserved, leading to the observed gaps between these assessments. Additionally, this study primarily involved children with spastic CP, which may have influenced the results. The relatively higher correlation observed with the MACS may be attributed to the assessment item in the EDACS related to mealtimes [6,14,15]. In this study, correlation with the MACS was the highest (Kτ = 0.741) among the other functional classification systems. This is relevant because the MACS reflects proficiency in tool use.

### 4.3. Limitations

This study primarily included relatively high-functioning children with CP, particularly those with proficient eating and drinking abilities. Among the children evaluated using the EDACS, only one was identified as level V based on caregiver assessment, aligning with the absence of children relying solely on tube feeding. Furthermore, in the first EDACS evaluation, 15 children (37.5% of the total) were assessed as level I by all three evaluators. However, distributions ranging from levels I to V were observed across the GMFCS, MACS, and CFCS. This highlights the need to consider the EDACS as distinct from other functions, specifically eating and drinking, based on findings in previous reports and clinically relevant findings [5,24]. Using video recordings to evaluate the EDACS was deemed methodologically favorable; however, conducting recordings at home may be difficult, especially if additional assistance during mealtime is required. This may explain why a greater number of children with better functioning based on the EDACS assessment were included as participants in this study.

Although children with EDACS level V were not excluded by design, all participants in this study were capable of oral feeding, and none were tube-dependent. As EDACS level V typically represents children who cannot eat or drink safely and require full tube feeding, this level was not represented in our sample. In addition, children with level IV were also underrepresented, resulting in an over-representation of higher-functioning children. This limits the generalizability of our findings to the broader population of children with CP, particularly those with more severe impairments. Future research should aim to include a more functionally diverse sample to better evaluate the reliability and applicability of the EDACS across the full spectrum of severity.

## 5. Conclusions

The Korean version of the EDACS has excellent reliability in evaluating eating and drinking abilities among children with CP. Thus, it serves as a valuable tool for enhancing communication among physicians, therapists, and caregivers within the field of rehabilitation medicine. Moreover, it shows close correlations with other functional assessment tools used for children with CP.

## Figures and Tables

**Table 1:** Demographic and clinical characteristics of the study participants.

Characteristics	
**Age at assessment (years), mean (range)**	8.4 (3–17)
**Gestational age (weeks), mean (range)**	32.5 (21–40)
**Sex, *n* (%)**	
Male	21 (52.5)
Female	19 (47.5)
**Tone abnormality**	
Spastic	30 (75.0)
Dyskinetic	2 (5.0)
Ataxic	3 (7.5)
Mixed	2 (5.0)
Non-classifiable	3 (7.5)
**Motor distribution, *n* (%)**	
Unilateral	6 (12.0)
Bilateral	44 (88.0)
**GMFCS, *n* (%)**	
I	12 (30.0)
II	9 (22.5)
III	4 (10.0)
IV	10 (25.0)
V	5 (12.5)
**MACS, *n* (%)**	
I	11 (27.5)
II	8 (20.0)
III	12 (30.0)
IV	4 (10.0)
V	5 (12.5)
**CFCS, *n* (%)**	
I	13 (32.5)
II	4 (10.0)
III	3 (7.5)
IV	2 (5.0)
V	18 (45.0)

GMFCS, Gross Motor Function Classification System; MACS, Manual Ability Classification System; CFCS, Communication Function Classification System.

**Table 2:** Agreement on the EDACS level between a physician, a speech–language therapist, and caregivers.

	Physician *					
SaLT *	I	II	III	IV	V	Total
I	22	1	0	0	0	23
II	0	3	0	0	0	3
III	0	1	5	1	0	7
IV	0	0	0	7	0	7
V	0	0	0	0	0	0
Total	22	5	5	8	0	40
	**SaLT ^†^**					
**Caregiver ^†^**	I	II	III	IV	V	Total
I	15	0	1	0	0	16
II	3	2	1	0	0	6
III	5	0	4	3	0	12
IV	0	1	1	3	0	5
V	0	0	0	1	0	1
Total	23	3	7	7	0	40
	**Caregiver ^‡^**					
**Physician ^‡^**	I	II	III	IV	V	Total
I	15	3	4	0	0	22
II	1	2	1	1	0	5
III	0	1	4	0	0	5
IV	0	0	3	4	1	8
V	0	0	0	0	0	0
Total	16	6	12	5	1	40

* κ = 0.940, 95%CI 0.874–1.006; *p* < 0.001. ^†^ κ = 0.557, 95%CI 0.374–0.740; *p* < 0.001. ^‡^ κ = 0.618, 95%CI 0.450–0.785; *p* < 0.001. EDACS: Eating and Drinking Ability Classification System; SaLT: speech and language therapist. **Note.** Background color indicates cells where both raters were in agreement.

**Table 3:** Agreement on EDACS level of assistance between a physician, a speech–language therapist, and caregivers.

	Physician *			
SaLT *	Independent	Requires assistance	Totally dependent	Total
Independent	20	3	0	23
Requires assistance	0	6	0	6
Totally dependent	0	1	10	11
Total	20	10	10	40
	**SaLT ^†^**			
**Caregiver ^†^**	Independent	Requires assistance	Totally dependent	Total
Independent	12	0	0	12
Requires assistance	11	6	5	22
Totally dependent	0	0	6	6
Total	23	6	11	40
	**Caregiver ^‡^**			
**Physician ^‡^**	Independent	Requires assistance	Totally dependent	Total
Independent	12	8	0	20
Requires assistance	0	10	0	10
Totally dependent	0	4	6	10
Total	12	22	6	40

* κ = 0.887, 95%CI 0.780–0.994; *p* < 0.001. ^†^ κ = 0.542, 95%CI 0.0.365–0.719; *p* < 0.001. ^‡^ κ = 0.636, 95%CI 0.461–0.812; *p* < 0.001. EDACS: Eating and Drinking Ability Classification System; SaLT: speech and language therapist. **Note.** Background color indicates cells where both raters were in agreement.

**Table 4:** Distribution of EDACS level compared with FOIS, GMFCS, MACS, and CFCS agreement.

	EDACS				
	**I**	**II**	**III**	**IV**	**Total**
**FOIS**					
IV	0	0	0	1	1
V	0	0	3	3	6
VI	1	3	2	4	10
VII	21	2	0	0	23
Total	22	5	5	8	40
**GMFCS**					
I	11	1	0	0	12
II	7	0	1	1	9
III	3	1	0	0	4
IV	1	3	3	3	10
V	0	0	1	4	5
Total	22	5	5	8	40
**MACS**					
I	10	1	0	0	11
II	8	0	0	0	8
III	4	3	2	3	12
IV	0	1	2	1	4
V	0	0	1	4	5
Total	22	5	5	8	40
**CFCS**					
I	13	0	0	0	13
II	3	1	0	0	4
III	21	1	0	0	3
IV	1	1	0	0	2
V	3	2	5	8	18
**Total**	22	5	5	8	40

EDACS, Eating and Drinking Ability Classification System; FOIS, Functional Oral Intake Scale; GMFCS, Gross Motor Function Classification System; MACS, Manual Ability Classification System; CFCS, Communication Function Classification System. **Note.** EDACS level V does not appear in the table because it was not assigned by either professional evaluator (physician or SaLT). All participants were capable of oral feeding (FOIS ≥ IV), and no child was fully tube-dependent.

**Table 5:** Distribution of the EDACS level of assistance compared with FOIS, GMFCS, MACS, and CFCS.

	EDACS			
	**Independent**	**Requires Assistance**	**Totally Dependent**	**Total**
**FOIS**				
IV	0	0	3	2
V	0	3	2	6
VI	1	4	4	9
VII	19	3	1	23
Total	20	10	10	40
**GMFCS**				
I	11	1	0	12
II	7	1	1	9
III	1	3	0	4
IV	1	5	4	10
V	0	0	5	5
Total	20	10	10	40
**MACS**				
I	11	1	0	12
II	7	0	0	7
III	2	8	2	12
IV	0	1	3	4
V	0	0	5	5
Total	20	10	10	40
**CFCS**				
I	11	2	0	13
II	3	1	0	4
III	2	1	0	3
IV	1	0	1	2
V	3	6	9	18
**Total**	20	10	10	40

EDACS, Eating and Drinking Ability Classification System; FOIS, Functional Oral Intake Scale; GMFCS, Gross Motor Function Classification System; MACS, Manual Ability Classification System; CFCS, Communication Function Classification System.

## Data Availability

The data supporting this study are not publicly available due to privacy concerns but can be obtained from the corresponding author upon request.

## Abbreviations

The following abbreviations are used in this manuscript:

EDACS	Eating and Drinking Ability Classification System
CP	Cerebral palsy
SaLT	Speech and language therapist
FOIS	Functional Oral Intake Scale
GMFCS	Gross Motor Function Classification System
MACS	Manual Ability Classification System
CFCS	Communication Function Classification System
